# Modeling of the irradiation effect on some physicochemical properties of metoprolol tartrate for safe medical uses

**DOI:** 10.1038/s41598-019-56805-0

**Published:** 2020-01-09

**Authors:** Najoua Ouerfelli, Narcisa Vrinceanu, Ezzedine Mliki, Abdelgadir M. Homeida, Kamal A. Amin, Magdalena Ogrodowczyk, Fawziah S. Alshehri, Noureddine Ouerfelli

**Affiliations:** 1 Université de Tunis El Manar, Faculté des Sciences de Tunis, Laboratoire de Matériaux, Cristallochimie et Thermodynamique Appliquée, 2092 El Manar II, Tunis, Tunisia; 2 “Lucian Blaga” University of Sibiu, Romania, Department of Industrial Machinery and Equipment, 10 Victoriei Boulevard, 550024 Sibiu, Romania; 3 Université de Tunis El Manar, Laboratoire Biophysique et de Technologies Médicales LR13ES07, Institut Supérieur des Technologies Médicales de Tunis, 9 Avenue Dr. Zouhaier Essafi, 1006 Tunis, Tunisie; 4 Departments of Mathematics, College of Science, Imam Abdulrahman Bin Faisal University, P.O. Box 1982, Dammam, 31441 Saudi Arabia; 5 Basic & Applied Scientific Research Center, Imam Abdulrahman Bin Faisal University, P.O. Box 1982, Dammam, 31441 Saudi Arabia; 6 Department of Biology, College of Science, Imam Abdulrahman Bin Faisal University P.O. Box 1982, Dammam, 31441 Saudi Arabia; 7 Department of Chemistry, College of Science, Imam Abdulrahman Bin Faisal University, P.O. Box 1982, Dammam, 31441 Saudi Arabia; 8 Department of Pharmaceutical Chemistry, K. Marcinkowski University of Medical Sciences, 60-780 Poznań, Grunwaldzka 6, Poland

**Keywords:** Biophysics, Natural hazards, Cardiology, Chemistry, Materials science, Mathematics and computing, Biological physics, Chemical physics, Statistical physics, thermodynamics and nonlinear dynamics

## Abstract

The effect of gamma-irradiation and ionizing radiation (high-energy electrons beam) on the physicochemical properties of metoprolol tartrate at the solid phase and aqueous solution, has been investigated in the present study to model some properties affected by absorbed doses and to reveal some interesting mutual causal correlation. The proposed some interesting models can be adapted to other experimental conditions, and the newly obtained values of the adjustable parameters could be an excellent criterion of the state quality of the metoprolol tartrate or for other additional interpretations. The peculiar behaviour of variation of physicochemical properties against dose leads us to confirm the suggested optimized doses mentioned in previous work, for sterilization and safe medical uses.

## Introduction

**Figure 1:**
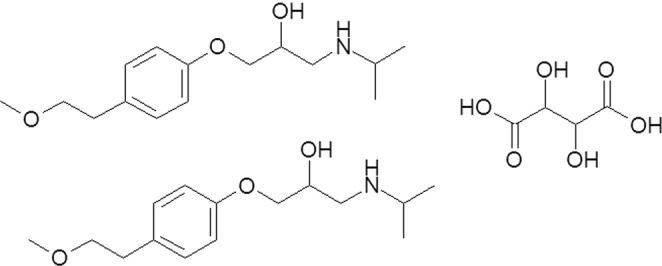
Metoprolol tartrate Structural formulae: 1-[4-(2-methoxyethyl)phenoxy]-3-[(1-methylethyl)amino]-2-propanol (2:1) dextro-tartrate salt.

As a synthetic *β*-1 adrenoceptor-blocking agent, the metoprolol tartrate (Fig. 1) is an antihypertensive drug and also used to treat different conditions such as high blood pressure, heart failure, and angina (chest pain)^1–3^. Moreover, the metoprolol tartrate is used in industrial sterilization by ionizing radiation (*β* and *γ* rays or by high-energy electrons beans)^1,2,4–6^. It was noticed that ionizing radiation works by energy transfer through the adsorption of this energy by the target materials^4–11^. Generally, the ionization of materials occurs at room temperature and the treatment depth varies with the nature and dose of radiation. Our works suggest that an excessive radiation dose can cause breaks in the chromosomal DNA of some micro-organisms at the biological cellular-level, which can lead to their damage or death.

In previous work, *γ*-irradiation (0–50 kGy) on metoprolol tartrate^1^ for which; thermal analysis (such as Thermogravimetric analysis (TGA) and Differential thermal analysis (DTA)), X-ray analysis, UV-analysis, IR spectra, and high-pressure liquid chromatography show good analysis.

As, the metoprolol tartrate has high resistance to *γ*–adsorbed doses (20 to 40) kGy and that’s why it conserves approximately its crystallinity. Moreover, this behaviour is also observed in the case of a high-energy electron beam irradiation^3^. It was concluded that this interval of doses can be utilized safely for metoprolol tartrate sterilization (ISO 11137) for special pharmaceutical and medical applications. In this work, we will try to give an optimal dose value between 20 and 40 kGy of *γ*-irradiation.

Similarly in previous work we have used high-energy electron beam irradiation (from 0 to 400 kGy) on metoprolol tartrate at solid phase^3^. The effect of irradiation dose has been inspected and tested by some analytical methods such as chromatography, UV and IR spectrophotometry and electron magnetic resonance (EPR). We concluded that the metoprolol tartrate presents good resistance to 25 kGy doses of electron-beam irradiation with safety. Nevertheless, the high doses of electrons-irradiation cause a change of some physicochemical properties such as the pH of metoprolol tartrate aqueous solutions, the melting point, the UV absorbance, the color and the content of water in metoprolol tartrate in a solid phase. Also, a high dose can induce radiolytic degradation, presence of moisture, formation of radiodegradation products, etc. Moreover, the radiostability of metoprolol tartrate aqueous solutions and the effect of the absorbed dose (between 0 and 50 kGy), dose rate (high-energy electron beam versus *γ*-irradiation) and radioprotectors (pharmaceutical excipients) are investigated through computer simulations and by HPLC-UV analyses^12^.

In the present work, we will try to model the effect of the two types of irradiation doses on some physicochemical properties of metoprolol tartrate in solid-phase or aqueous solutions^12–21^ in order to predict and estimate some experimental results when some parameters are not available. We will suggest a value of optimization dose for safe medical and pharmaceutical uses, sterilization, hypertensive treatments, etc.

It was noted that the present work (as the first in the biochemical systems) comes in the general framework of a general modelling project for some physicochemical properties in different disciplines of applied chemistry^22,23^, in order to allow possible prediction or estimation of certain properties, or to use the obtained values of some adjustable parameters as criterion or diagnostic factors in certain practical uses.

## Effect on *γ*-irradiation on Metoprolol Tartrate

### Experimental

Experimental details are presented in the previous work^1^ where a set of metoprolol tartrate samples were subjected to γ-irradiation at 30 cm distance, using Cs-137 source for absorbed doses from 0 to 50 kGy.

X-ray manipulations were made using *γ*-irradiated metoprolol tartrate in solid phase, while in UV-Absorption and some thermal study (such as Thermogravimetric analysis (TGA) and Differential thermal analysis (DTA)) of metoprolol tartrate samples were dissolved in anhydrous ethanol solutions with fixed concentration (20 mg/mL) in different *γ*-absorbed doses (from 0 to 50 kGy)^1^. More details are given in the previous work^1^.

### Irradiation dose and crystallinity of metoprolol tartrate

Starting from the X-ray diffraction analysis of metoprolol tartrate before and after *γ*-irradiation doses presented in the previous work^1^, we have inspected the variation of all peak intensities of X-ray at different absorbed doses (from 0 to 50 kGy) presented in X-ray patterns.

**Figure 2:**
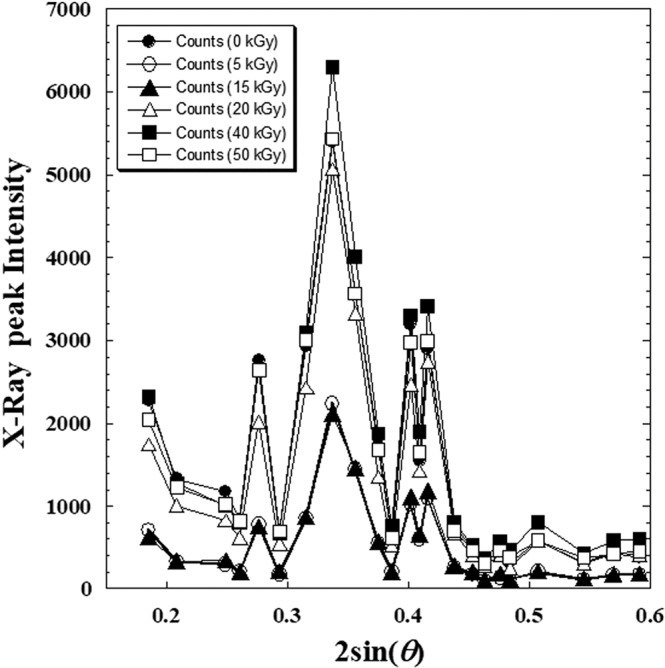
Superposition of X-ray diffraction patterns of metoprolol, β-blocker^1^; (●): before radiation, and the rest after γ-irradiation doses; (○): 5 kGy dose; (▲): 15 kGy dose; (∆): 20 kGy dose; (■): 40 kGy dose and (□): 50 kGy dose.

In Fig. 2, only all peak intensities versus 2sin(*θ*) were interpolated. It was observed that a perfect similarity for which the intensities vary in the same sense when the dose value changes (i.e. from a diffractogram to another, there is only a shift in *y*-axis and not in *x*-axis).

**Figure 3:**
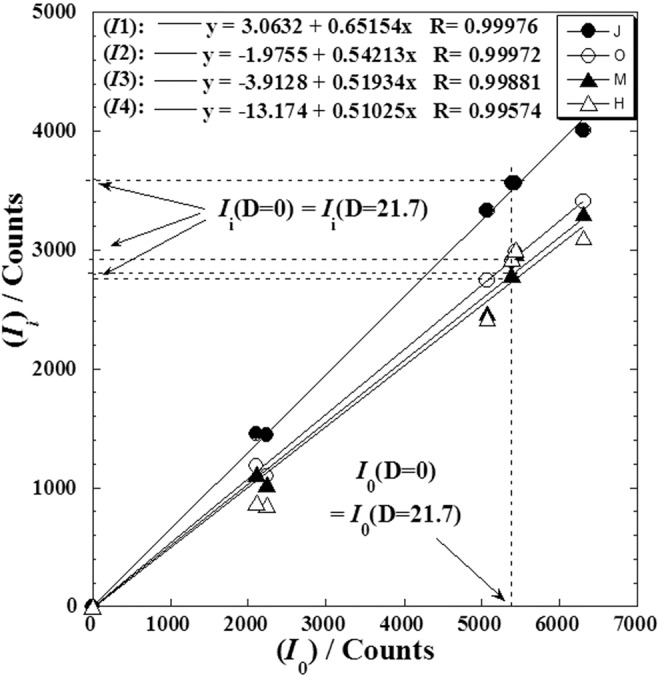
Causal correlation between intensities of the first four high intensive peaks (*I*_*i*_) and that of the strongest peak (*I*_0_) for different γ-irradiation doses varying from (0–50 kGy). (●): (*I*_1_); (○): (*I*_2_); (▲): (*I*_3_) and (∆): (*I*_4_). (*I*_1_) > (*I*_2_) > (*I*_3_) > (*I*_4_). The symbols J, M, O and N designate the picks number in Table 1.

For illustrating this behaviour, we correlated the intensities (*I*_*i*_) of the first four high intensive peaks to that of the strongest peak (*I*_o_) for different *γ*-irradiation doses (Fig. 3) varying from 0 to 50 kGy.

A reliable linear dependence between the intensities (*I*_*i*_) of four peaks and that of the strongest peak (*I*_0_) with an average of correlation coefficient (*R* = 0.99867) was observed. Neglecting the value of the intercept on the ordinate (see linear equation into Fig. 3) in front of the value of intensities (*I*_*i*_), the correlation can be expressed as follows:

1 $${y}_{i}={a}_{i}x$$

where (*x*) is the intensity of strongest peak (*I*_o_) at a given dose (*D*), (*y*_*i*_) is the intensity of one of the four high intensive peaks (*I*_*i*_; *i* = 1,2,3,4) at the same given dose (*D*) and (*a*_*i*_) at the coefficient of proportionality giving the relative intensity. We conclude that the (*a*_*i*_)-coefficient is dependent on the irradiation dose *D* and the relative intensity *I*_*i*_(*D*)/*I*_o_(*D*) is conserved weather the value of (*D*).

**Figure 4:**
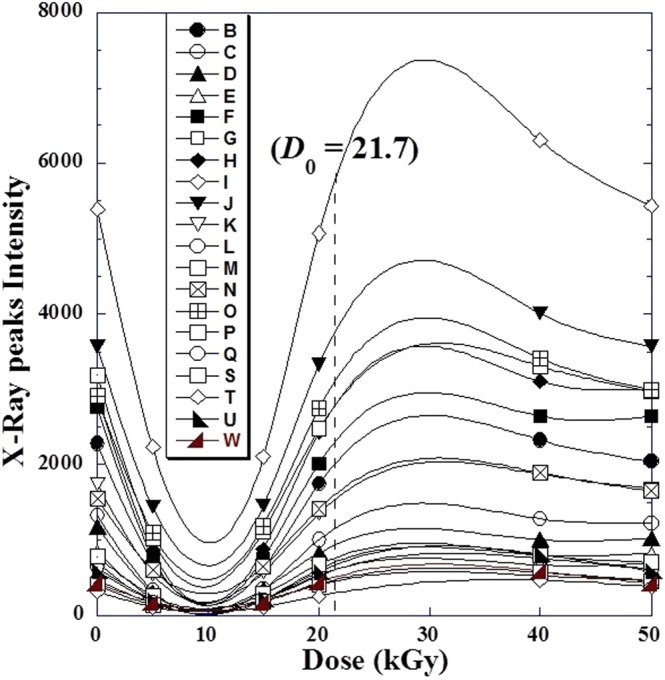
Similarity of variation of different X-ray peaks intensity (*I*_*i*_) with γ-irradiation doses varying from (0–50 kGy) by metoprolol. Each capital letter corresponds to a given 2sin(*θ*)-value X-ray patterns ant it is indicated in Table 1.

However, inspecting the variation of each peak’s intensity (*I*_*i*_) for the whole X-ray diffractogram with irradiation dose (*D*) for metoprolol tartrate (Fig. 4) and (Table 1), we refined in another manner the observed similarity in Fig. 2.

**Table 1:** X-ray diffraction analysis of metoprolol tartrate before (0 Gy) and after *γ*-irradiation doses (5 to 50 kGy).

2sin(*θ*)	Symbol	*γ*-irradiation dose/ kGy					
		0	5	15	20	40	50
		1/*d* (Å^−1^)					
0.18561	B	0.12054	0.12071	0.12048	0.11997	0.12127	0.12054
0.20906	C	0.13570	0.13559	0.13587	0.13520	0.13655	0.13546
0.24893	D	0.16164	0.16227	0.16175	0.16125	0.16265	0.16192
0.26105	E	0.16973	0.16990	0.16945	0.16912	0.17052	0.16939
0.27662	F	0.17961	0.17972	0.17950	0.17900	0.18040	0.17954
0.29389	G	0.19108	0.19178	0.19144	0.19071	0.19212	0.19150
0.31580	H	0.20499	0.20460	0.20493	0.20426	0.20549	0.20493
0.33698	I	0.21924	0.22013	0.21896	0.21835	0.22047	0.21952
0.35589	J	0.23214	0.23185	0.23112	0.23106	0.23224	0.23106
0.37511	K	0.24366	0.24321	0.24310	0.24254	0.24382	0.24337
0.38676	L	0.25188	0.25221	0.25244	0.25088	0.25199	0.25183
0.40198	M	0.26093	0.26082	0.26093	0.26038	0.26166	0.26088
0.40968	N	0.26593	0.26587	0.26598	0.26520	0.26654	0.26576
0.41582	O	0.27069	0.27020	0.27042	0.26964	0.27096	0.27047
0.43816	P	0.28371	0.28431	0.28458	0.28392	0.28552	0.28469
0.45330	Q	0.29491	0.29474	0.29458	0.29386	0.29502	0.29441
0.46333	R	0.30003	0.30097	0.30097	0.30047	0.30114	0.30099
0.47588	S	0.30814	0.30893	0.30928	0.30807	0.30862	0.30876
0.48401	T	0.31434	0.31450	0.31649	0.31457	0.31495	0.31556
0.50752	U	0.33010	0.32840	0.32938	0.32879	0.32944	0.32949
0.54540	V	0.35314	0.35327	0.35364	0.35403	0.35359	0.35469
0.56970	W	0.37001	0.37160	0.37028	0.36997	0.37110	0.37097
0.59142	X	0.38454	0.38393	0.38440	0.38405	0.38682	0.38569

**Figure 5:**
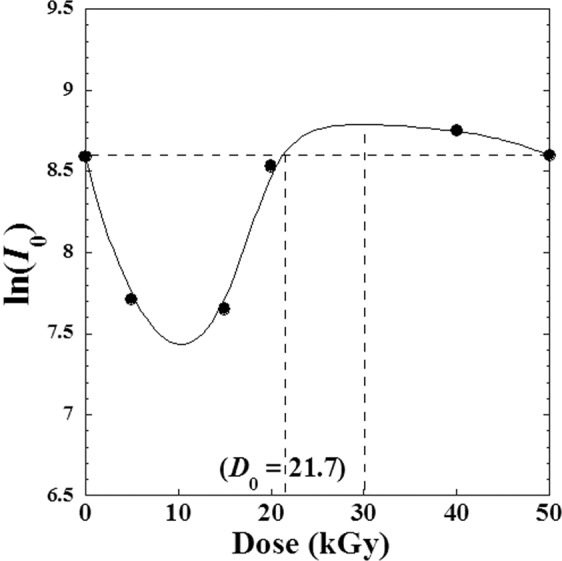
Variation of the natural logarithm of the intensity the strongest peak (I0) for different γ-irradiation doses (D) varying from (0–50 kGy) for metoprolol.

In addition, we can interpret the increase of the peak intensity *I*_*i*_(*D*) with dose (*D*) indicates the increase of material degree of crystallinity. Figure 5 shows the variation of the strongest peak intensity *I*_0_(*D*) against the absorbed dose (*D*). We can see that *I*_0_(*D*) decreases until the *D*≈10 kGy and increases to reach a maximum at *D* ≈ 30 kGy and then decreases where material undergoes back partial dissociation^1^ until 40 kGy.

In the previous work^1^ it was concluded that the metoprolol tartrate preserves a high resistance to *γ*-absorbed dose (*D*) between 20 and 40 kGy and can be used safely for sterilization. So, we observe that the peak intensity *I*_0_(*D*) refined its initial value before irradiation at approximately *D* = 21.7 kGy where the variation show a change of curvature.

From this fact we can think about the choice of *D*_0_ = 21.7 kGy as an optimized dose (20 < *D*_0_ < 40) where the material identity is still well preserved.

We observed also, quasi-equality between the intensity of any peak before dose *I*_*i*_(*D* = 0) and that after irradiation by optimal dose *I*_*i*_(*D*_0_ ≈ 21.7). This could be easily seen in Fig. 4 when we plot a tangent going through the initial value at (*D* = 0), which intercept any curve approximately at (*D*_0_≈21.7). The same ascertainment is clearly observed by some projections in Fig. 3 for the first four high intensive peaks (*I*_*i*_).

**Figure 6:**
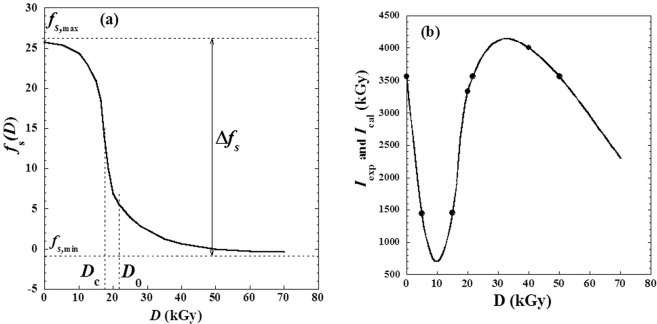
(**a**) Variation of specific function *f*_*S*_(*D*) (Eq. 3) and (**b**) comparison between experimental data of X-ray *i*-peak intensity *Ii*(*D*) and that calculated by the suggested Eq. (2).

Regarding this behaviour, an empirical model correlating the variation of the intensity *I*_*i*_(*D*) of any peak (*i*) versus the absorbed *γ*-irradiation dose (*D*) could be suggested as follows:

2 $${I}_{i}(D)={I}_{i}(D=0)+D\cdot (D-{D}_{0})\cdot {f}_{Si}(D)$$

where *f*_*Si*_(*D*) is a specific function characterizing the (*i*)^th^ peak (Fig. 6a). Note that experimental data and calculated values are in excellent agreement (Fig. 6b).

Moreover, the specific function *f*_*Si*_(*D*) can be extrapolated for doses (*D*) greater than 50 kGy with a reliable accuracy (because of the horizontal asymptote in Fig. 6a). So, from this fact it could be predicted with good certainty that the intensity value *I*_i_(*D*) continue to decrease for high doses (*D*) greater than 50 kGy (Fig. 6a) and affirm that these values are not suitable for medical and pharmaceutical safe uses we add that due to similarity of the curve-shape of specific function *f*_*Si*_(*D*) with the Fermi-Dirac distribution, we suggested an interesting empirical expression correlating *f*_*Si*_(*D*) against dose (*D*) as follows:

3 $${f}_{Si}(D)=\frac{({f}_{S,max}-{f}_{S,min}){e}^{-\alpha (D-{D}_{c})}}{1+{e}^{-\alpha (D-{D}_{c})}}$$

where *f*_*S*,max_ and *f*_*S*,min_ are the values of limits of the function *f*_*Si*_(*D*) characterizing by the two horizontal asymptotes indicated in Fig. 6a (i.e. the absolute maximum and minimum of the function *f*_*Si*_(*D*)), (*D*_*c*_) can be called critical dose which accurate approximately at the inflection point, and (*α*) is a factor depending of temperature and the nature of the interaction between *γ*–photons and the material particles. Moreover, if the physics dimensional equation was applied, we can affirm that the reciprocal (*α*)-value is an interesting characteristic dose (δ = 1/*α*) which can be used for interpretation and discussion in relationship with the specificity of the nature of the studied system and the experimental conditions.

Furthermore, in the case of other experimental conditions and similar *γ*-irradiation on metoprolol tartrate, we could apply Eq. (3) in Eq. (2) and let all parameters (*D*_0_, *D*_*c*_, *α*, *f*_*S*,max_ and *f*_*S*,min_) in non-linear regression as free adjustable ones; interesting specific values for the new used experimental conditions will be obtained.

From experimental data presented in previous work^1^ on X-Ray diffraction analysis of metoprolol tartrate before and after *γ*-irradiation doses (*D*), the reciprocal of *d*_*hkl*_ and 2sin(*θ*) was calculatd in order to test the agreement with the Braag’s law (Eq. 4) for n = 1 at each given absorbed dose (*D*)^1,24,25^.

4 $$2{d}_{hkl}\,\sin (\theta )=n\lambda $$

**Figure 7:**
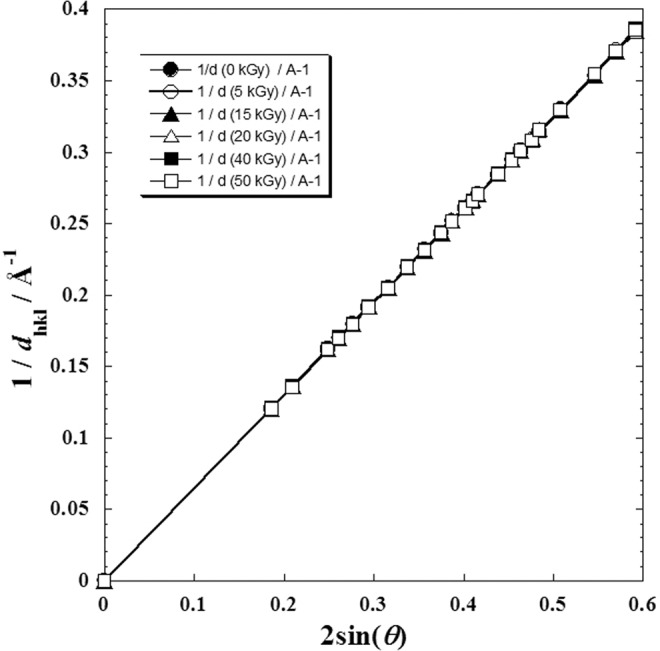
Mutual dependence between the two Bragg’s parameters (*d*_hkl_ and *θ*) at absorbed dose (D) by metoprolol, *β*-blocker; (●): before radiation, and the rest after γ-irradiation doses; (○): 5 kGy dose; (▲): 15 kGy dose; (∆): 20 kGy dose; (■): 40 kGy dose and (□): 50 kGy dose.

The plot of the reciprocal of *d*_*hkl*_ against 2sin(*θ*) (Fig. 7) leads us to conclude that the metoprolol tartrate well conserves its crystalline structure for each dose (*D*). From the slope of each straight line, the value of the wavelenght (*λ*) for each absorbed dose (Table 2) with an excellent correlation coefficient *R* = 0.99999 could be deduced.

**Table 2:** Variation of some parameters related to X-ray diffraction, UV spectrophotometry and thermal analysis against different *γ*-irradiation doses (*D*) varying from (0 to 50) kGy for metoprolol tartrate.

*D*	*λ*	UV Absorbance	*I*_0_(*D*)/*I*_0_ (*D* = 0)	Δ*H*_1_	Δ*H*_2_
kGy	Å	—	—	(kJ/g)	(kJ/g)
0	1.54029	0.285	1.000	34.60	116.9
5	1.53998	0.281	0.396	141.9	141.9
15	1.53856	0.295	0.335	102.7	81.80
20	1.54121	0.332	0.940	132.0	144.0
40	1.53714	0.309	1.170	108.0	130.0
50	1.53721	0.345	1.008	119.3	115.7

**Figure 8:**
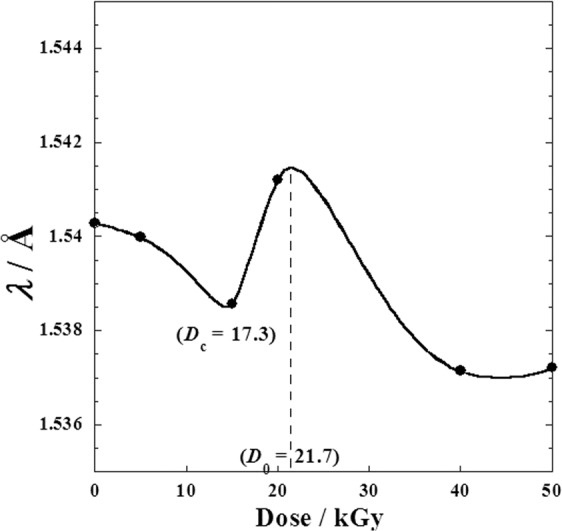
Variation of the X-ray wavelength *λ* (Å) against different *γ*-irradiation doses (*D*) varying from (0–50 kGy) for metoprolol.

Figure 8 shows a feeble variation of ***λ***-values with the irradiation dose (*D*) and a local maximum of 1.5472 Å at the suggested optimal dose *D*_0_ = 21.7 kGy.

**Figure 9:**
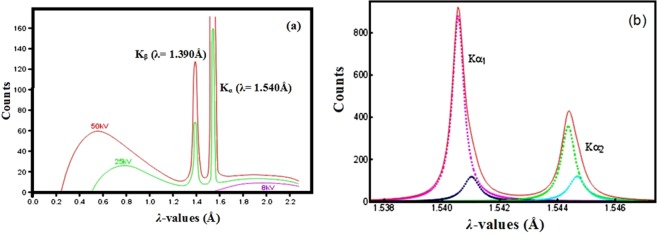
Source spectrum of double-crystal spectrometer measured CuK*α*_1,2_ spectrum and fitted Lorentzians.

It was noted that the X-ray diffraction patterns were performed in previous work^1^ with Philips analytical X-ray BV. Diffractometer type pw\1710 BASED using anode Cu-*K*α tube. Copper is the one most often used for proteins since it is hard, an efficient conductor of heat and the Cu*K*α emission is relatively intense. The wavelength of the X-rays produced is 1.540 Å (Fig. 9a). Nevertheless, the X-ray source doesn’t emit a perfect monochromatic wave (i.e. a Dirac peak), but as described in the literature, the measured *K*α_1_spectrum is well represented symmetric Lorentzians (Fig. 9b) to an *R*-factor of 1.3^24,25^.

That’s why the *λ*-values vary slightly (Fig. 8, Table 2) around a central value approximately 1.5392 Å ± 0.14% (i.e. ± 0.0022 Å) approximately, which it’s in agreement with the R-X *K*α_1_ source spectrum fitted in literature^24,25^.

### Effect of γ-irradiation dose on UV-absorption and thermal properties

**Figure 10:**
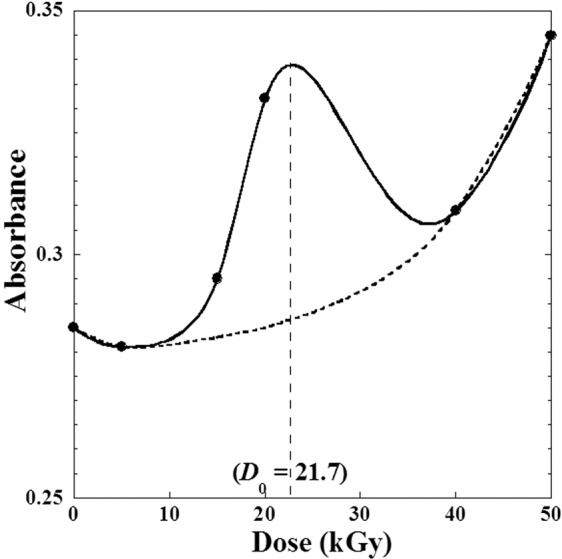
Variation of UV-electron absorbance against the γ-irradiation doses (*D*) varying from (0–50 kGy) for metoprolol. Dashed line represents interpolation without considering the effect of the optimization dose (*D*_0_).

For γ-irradiation manipulations, all the samples of metoprolol tartrate in anhydrous ethanol solutions were prepared with fixed concentration (20 mg/mL) in the whole range of *γ*-absorbed doses (from 0 to 50 kGy) and the UV-measurements are occurred with the wave length of *λ* = 220nm^1^. Table 2 and Fig. 10 show the variation of UV-electron absorbance with the γ-irradiation doses using the same wavelength (*λ*) for all samples which indicated the irradiated material preserves its identity^1^.

Nevertheless, though the variation of the optical density of the UV-spectra displays the same trend as X-Ray and IR data at different doses^1^, we observe globally a slight decrease of UV-absorbance with *γ*-dose characterizing by a local maximum at a suggested optimized dose (*D*_0_ = 21.7 kGy) approximately (Fig. 10).

The small decrease using a weak dose (0 < *D*_1_ = 6 kGy) indicates a probable dissociation due to the effect of *γ*–irradiation. While the increases of the UV-absorption be explained by the recombination of free radical and the crystallinity quality induced by the γ–irradiation doses^1^.

In the thermal studies (TGA and DTA) we detected a small variation of the melting points and weak weight loss^1^which indicates that the thermal behaviour preserves the identity of metoprolol tartrate after *γ*–irradiation which stabilizes it with a slight change^1^.

**Figure 11:**
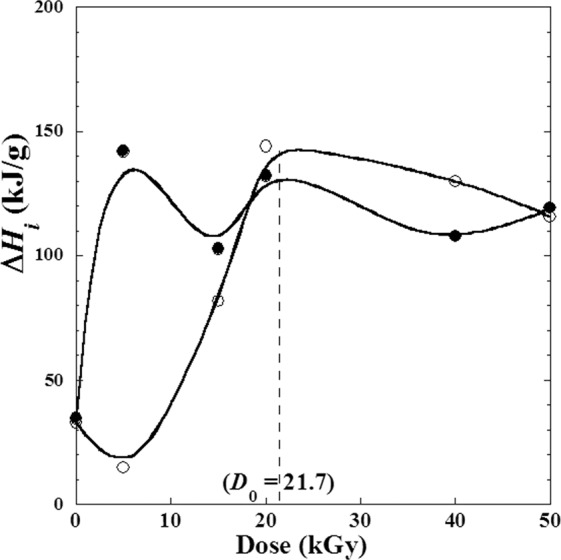
Variation of the enthalpies (Δ*H*_*i*_) determined by (TGA, DTA) techniques against the *γ*-irradiation doses (*D*) varying from (0–50 kGy) for metoprolol. (●): melting of metoprolol; (○): stepwise oxidative decomposition of metoprolol.

Nevertheless, though the globally conservation of metoprolol tartrate structure, we observe (except the first value of Δ*H*_1_) small variation of the heat enthalpy (∆*H*_1_) as a result of melting and the heat enthalpy (∆*H*_2_) due to the stepwise oxidative decomposition of metoprolol tartrate (Table 2, Fig. 11) showing the apparition of some new products in small quantities and due to a weak degradation. We observed that ∆*H*_2_ occurs in local minimum at *D*_1_ = 6 kGy and local maximum at the suggested optimized dose *D*_0_ = 21.7 kGy, while ∆*H*_1_ shows two local maxima at the two doses *D*_1_ and *D*_2_.

## Effect of the Electron-Radiation on Metoprolol Tartrate

### Experimental

Experimental details are presented in the previous work^3^ where metoprolol tartrate in solid phase was irradiated by high-energy electrons beam using an accelerator at doses varying from 0 to 400 kGy, while the obtained experimental *pH*-values were measured in aqueous solutions formed by 0.1500 g of metoprolol tartrate, irradiation at different doses (*D*) and then dissolved in 3 mL of distilled water^3^. More details are given in previous work^3^.

### Effect of absorbed doses on pH and melting point of metoprolol tartrate

**Figure 12:**
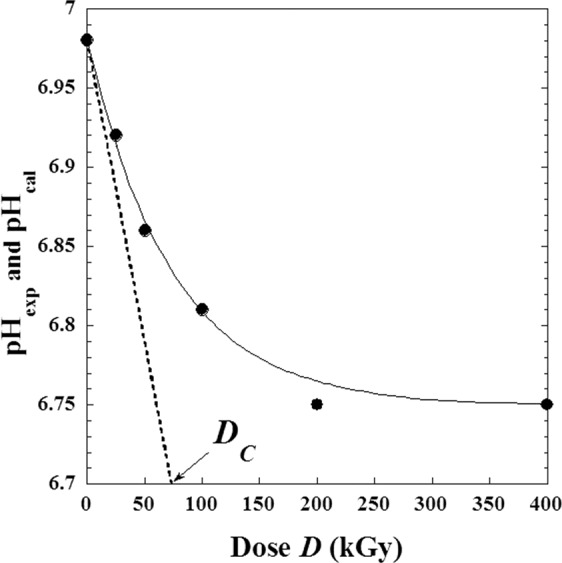
Experimental values of *pH* solutions of metoprolol with dose (*D*) of high-energy electron beam, (continued line): calculated pH-values by the suggested Eqs. 5 and 6.

The pH of these solutions decreases slightly following an exponential dependence with doses (*D*) and it can be expressed as follows:

5 $$pH=(p{H}_{0}-p{H}_{\infty })\cdot {e}^{-D/{D}_{C}}+p{H}_{\infty }$$

where *pH*_0_ is the initial value before irradiation i.e. *pH*_0_ = *pH*(*D*_0_) = 6.98, *pH*_∞_ is the final value at very high irradiation dose and *D*_*C*_ is an adjustable parameter (*D*_*C*_ = 73.4 kGy) equivalent to a dose and it is graphically determined by the intercept on the abscises a axis of the half tangent on initial point (Fig. 12).

For the experimental conditions used in the previous work^3^, we found numerically the following expression:

6 $$pH(D)=0.23\cdot \,{e}^{\frac{-D}{73.4}}+6.75\,$$

With a regression correlation coefficient *R* = 0.99755. Note that adjustable parameter values are obtained according to specific experimental conditions that can change for other situations and can be also an indicator for the same protocols or diagnostics in medical and pharmaceutical uses.

**Figure 13:**
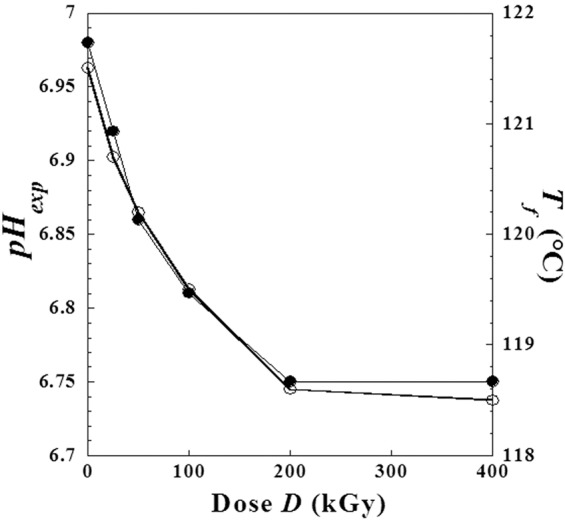
Similarity of behavior between the *pH* of metoprolol solution and the melting point (*T*_*f*_) of metoprolol at solid phase against the dose (*D*) of high-energy electron beam. (●): *pH* and (○): *T*_*f*_.

**Figure 14:**
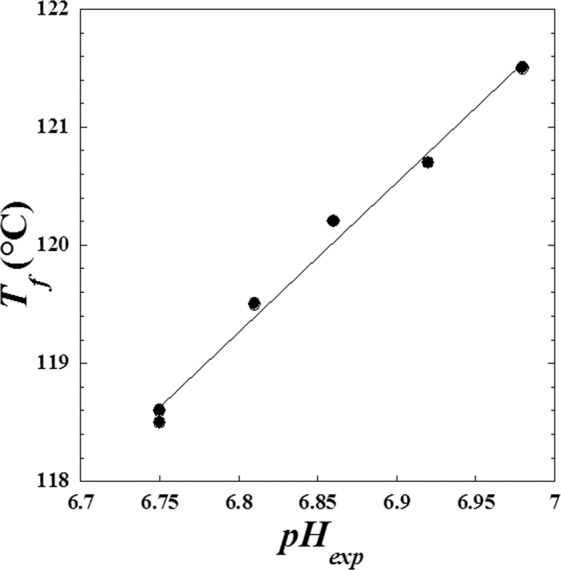
Mutual correlation between the *pH* of metoprolol solution and the melting point (*T*_*f*_) of metoprolol at solid phase when the absorbed dose (*D*) varies from (0 kGy to 400 kGy).

However, inspecting the decrease of melting point (*T*_*f*_) of the metoprolol tartrate with the absorbed doses *D* (Fig. 13) it was observed similar behaviour with the decrease of *pH* of metoprolol tartrate solution used in the previous work^3^. We conclude that there is probably a causal correlation revealed by Fig. 14.

Linear regression gives the following relationship:

7 $${T}_{f}(D)=32.83+12.71\cdot pH(D)$$

With a correlation coefficient equivalent to *R* = 0.99505. Note we have used as a melting point *T*_*f*_(*D*), approximately the center of each interval given in the previous study^3^.

According to precedent correlation, we can give similar model for the variation of melting point versus the dose which is expressed as follows:

8 $${T}_{f}(D)=({T}_{f,0}-{T}_{f,\infty })\cdot {e}^{\frac{-D}{{D}_{c}}}+{T}_{f,\infty }$$

where *T*_*f*,0_ is the melting point for metoprolol tartrate before irradiation, while *T*_*f*,∞_ is the final quasi-constant value at a very high irradiation dose.

Due to the precedent similarity, we note that the *Dc*-value is the same one obtained for *pH*, and can be called critical dose which can vary for different experimental conditions and also can be an indicator of contamination degree with the radiolysis products, etc.

### Effect of absorbed doses on water content and UV-absorption

**Figure 15:**
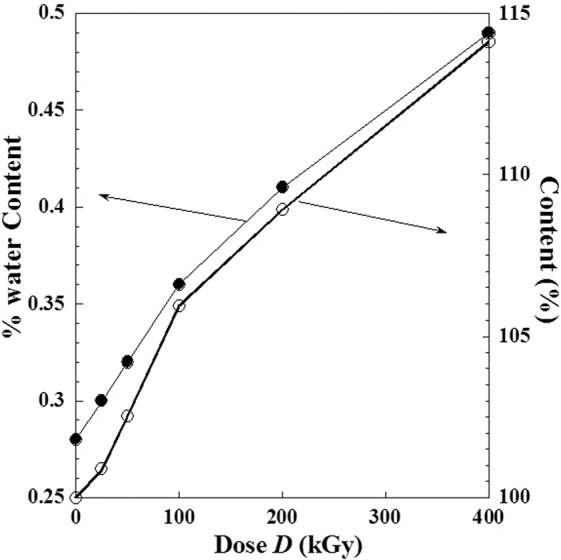
Similarity behavior between the (% water content) in metoprolol and the (UV- content %) against the dose (*D*) of high-energy electron beam. (●): % water content; (○): UV- content %.

**Figure 16:**
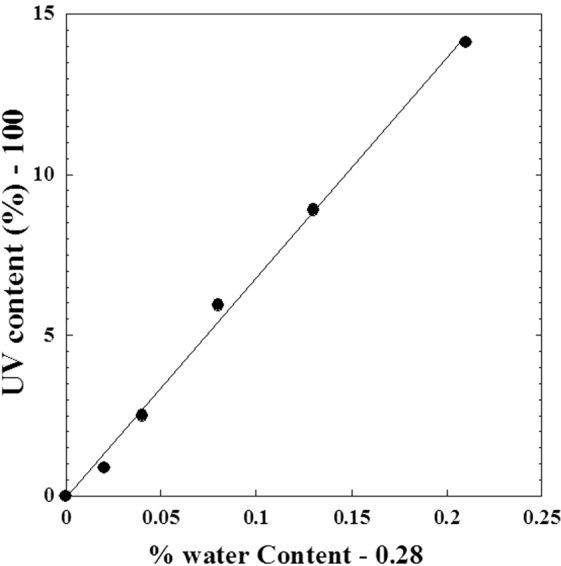
Mutual correlation between the (% water content) in metoprolol and the (UV- content %) when the absorbed dose (*D*) varies from (0 kGy to 400 kGy).

Comparison at different irradiation doses, between the content of water % in metoprolol tartrate in the solid phase and UV contents (%) of absorbance of metoprolol tartrate aqueous solution (irradiated at solid-state) is presented in the previous work^3^. Figure 15 shows similar behaviour between these two properties with the absorbed dose (*D*). It can be confirmed by the linear mutual dependence between the corresponding deviations to the state before irradiation which is showed by Fig. 16 and expressed by Eq. (9) obtained by linear regression fit with a slope equal to 68.243, a shift of water content equal to 0.28% and a correlation coefficient equal to *R* = 0.9987.

9 $${\rm{UV}}\,{\rm{content}}\,( \% )-100=68.243\times ( \% {\rm{water}}\,{\rm{content}}-0.28)$$

The obtained proportionality between the two deviations to values before irradiation can lead us to conclude that we can use only one of these properties as a criterion of an apparition of radio-degradation products with a similar structure with the parent compound.

## Conclusion

Results of X-ray and some thermal analyses, UV analysis, IR spectra, and high-pressure liquid chromatography presented in the previous study^1^, taken together with the present study indicating that metoprolol tartrate, possess high resistance to γ-absorbed doses (between 20 and 40) kGy all over a *γ*-induced high degree of crystallinity. We have concluded that the absorbed dose (between 20 and 40) kGy could be used safely for sterilization of metoprolol tartrate for medical and pharmaceutical applications. In the present work, we have shown that the metoprolol tartrate conserves a high degree of crystallinity and we have suggested semi-empirical equations relating X-ray peak intensity at different absorbed *γ*-irradiation dose.

In the same context, we have modelled the mutual strong correlation between the effects of absorbed doses on *pH* and melting point of metoprolol tartrate and it will be interesting if test in other experimental conditions to evaluate the existence of eventual effective causal correlation by taking the corresponding parameters as a free adjustable one and the new obtained values of the adjustable parameters. Such findings will be an excellent criterion of the state quality of the metoprolol tartrate or for other additional interpretations.

In addition, we have studied the effect of γ-irradiation dose on UV-absorption and some thermal properties and shown that at the suggested optimized dose (*D*_0_ = 21.7 kGy), the UV-electron absorbance and the enthalpies related to the thermal study present particular behaviour.

In the previous work^3^, metoprolol tartrate in solid-phase shows a resistant to ionizing radiation (high-energy electrons beam) used in the standard sterilization dose (25 kGy), therefore it was suitable for decontamination and sterilization by electron irradiation. So, in the present work, we suggested an interesting semi-empirical model describing the effect of the electron irradiation doses on some physicochemical properties of metoprolol tartrate in solid phase or in aqueous solutions. Moreover, the obtained critical dose value (*D*_*C*_ = 73.4 kGy) can change for other experimental conditions and it should be an interesting criterion to choose the optimization dose for safe medical and pharmaceutical uses, sterilization, hypertensive treatments, etc.

In the same context, we have modelled the mutual correlation between the effects of absorbed doses on water content and UV-absorption in our specific experimental conditions described above and in previous work^3^. This correlation should be tested in other experimental conditions to conclude if these findings, could be generalized.

## Data Availability

The data supporting the conclusions of this article are included within the article.
